# In silico re-identification of properties of drug target proteins

**DOI:** 10.1186/s12859-017-1639-3

**Published:** 2017-05-31

**Authors:** Baeksoo Kim, Jihoon Jo, Jonghyun Han, Chungoo Park, Hyunju Lee

**Affiliations:** 1 Gwangju Institute of Science and Technology, 123 Cheomdangwagi-ro,Buk-gu, Gwangju, 61005 Republic of Korea; 2 Chonnam National University, 77 Yongbong-ro, Buk-gu, Gwangju, 24105 Republic of Korea

**Keywords:** Drug target, Bioinformatics, Proteomics

## Abstract

**Background:**

Computational approaches in the identification of drug targets are expected to reduce time and effort in drug development. Advances in genomics and proteomics provide the opportunity to uncover properties of druggable genomes. Although several studies have been conducted for distinguishing drug targets from non-drug targets, they mainly focus on the sequences and functional roles of proteins. Many other properties of proteins have not been fully investigated.

**Methods:**

Using the DrugBank (version 3.0) database containing nearly 6,816 drug entries including 760 FDA-approved drugs and 1822 of their targets and human UniProt/Swiss-Prot databases, we defined 1578 non-redundant drug target and 17,575 non-drug target proteins. To select these non-redundant protein datasets, we built four datasets (A, B, C, and D) by considering clustering of paralogous proteins.

**Results:**

We first reassessed the widely used properties of drug target proteins. We confirmed and extended that drug target proteins (1) are likely to have more hydrophobic, less polar, less PEST sequences, and more signal peptide sequences higher and (2) are more involved in enzyme catalysis, oxidation and reduction in cellular respiration, and operational genes. In this study, we proposed new properties (essentiality, expression pattern, PTMs, and solvent accessibility) for effectively identifying drug target proteins. We found that (1) drug targetability and protein essentiality are decoupled, (2) druggability of proteins has high expression level and tissue specificity, and (3) functional post-translational modification residues are enriched in drug target proteins. In addition, to predict the drug targetability of proteins, we exploited two machine learning methods (Support Vector Machine and Random Forest). When we predicted drug targets by combining previously known protein properties and proposed new properties, an F-score of 0.8307 was obtained.

**Conclusions:**

When the newly proposed properties are integrated, the prediction performance is improved and these properties are related to drug targets. We believe that our study will provide a new aspect in inferring drug-target interactions.

**Electronic supplementary material:**

The online version of this article (doi:10.1186/s12859-017-1639-3) contains supplementary material, which is available to authorized users.

## Background

With the rapid accumulation of drug-related data in public databases, much attention has been paid to developing computational approaches to identify new drug candidates and to reposition existing drugs because computational tools help reduce time and costs of drug development [1]. Along with drug-related data, significant increases in proteomics data encourage researchers to focus on computational approaches in drug development. Similarities in amino acids sequences with existing drug targets and in functional roles of target proteins, including G-protein-coupled receptors (GPCRs), enzymes, and ion channels, have been main resources for inferring drug-target interactions, and many predictions have been performed within each functional category [2]. Recently, more resources, including side effects of drugs, drug-drug interactions, and protein-protein interactions, have been incorporated for predicting new drug targets [3, 4].

Such prediction efforts will be advanced if more properties of drug targets can be revealed. Over the last two decades, there have been several efforts to curate drug targets and to categorize them [5–8]. When Hopkins and Groom [5] identified 399 non-redundant molecular targets, targets were contained in only 130 protein families, half of which fall into just six gene families, including GPCRs and serine/threonine and tyrosine protein kinases. At that time, they predicted that the numbers of druggable genomes and drug targets would be approximately 3,000 and around 600-1500, respectively. Imming et al. [6] listed 218 targets and classified them based on “mechanism of actions", such as enzymes, substrates, metabolites, proteins, receptors, ion channels, transport proteins, DNA, RNA, ribosome, and targets of monoclonal antibodies. Recently, information about drugs and their targets have been systematically deposited in public databases. The DrugBank database [9], launched in 2006, is a systematic collection of drug-protein interactions containing information on more than 760 Food and Drug Administration (FDA)-approved drugs and around 2000 drug target proteins. Moreover, this database contains drug-target interactions with gene annotations from Swiss-Prot [10].

With the availability of various proteomics data, more comprehensive analysis about drug targets has become possible. Bakheet and Doig [11] defined 148 proteins as drug targets from the DrugBank database to analyze the protein target properties. They identified several features to distinguish targets from non-targets: all amino acid compositions, the length of proteins, hydrophobicity, secondary structure of proteins, transmembrane helices, and others. Bull and Doig [12] extended protein properties from Bakheet and Doig by proposing additional properties: protein-protein interactions, expression levels, and germline variants. However, these features were not strong indicators for distinguishing targets from non-targets. They also applied machine learning approaches such as support vector machine (SVM) and random forest (RF) to predict drug target proteins [11–13].

**Fig. 1:**
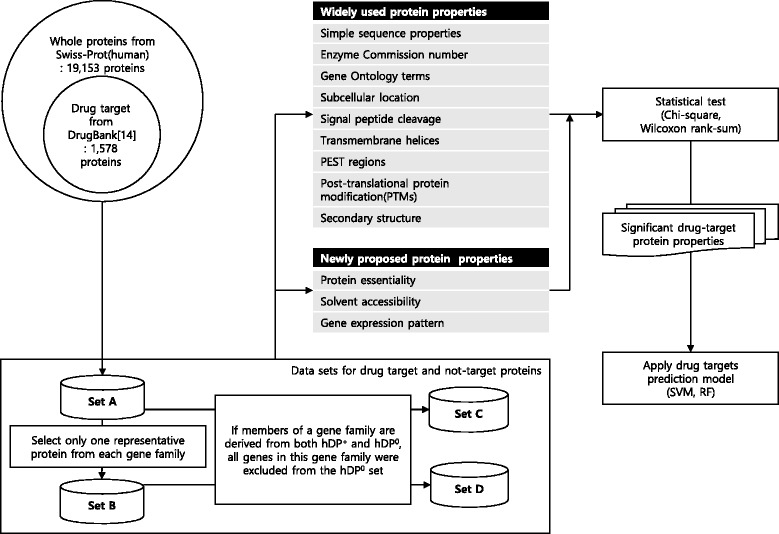
Flowchart of study design

Here, we explore more protein properties favorable to drug targets. Figure 1 shows our study design. We first made a protein list and then distinguished drug target proteins and non-target proteins. We then re-evaluated the protein properties used in Bakheet and Doig [11] by analyzing an increased number of drugs and targets in the DrugBank. For some properties, we employed manually curated datasets or multiple computational tools to estimate protein properties more reliably. We then showed that novel protein properties, including gene essentiality, gene expression levels, tissue specificity, and solvent accessibility, have different characteristics between targets and non-targets with statistical significance. Finally, we predicted drug targets based on these properties using SVM and RF and evaluated prediction accuracies. We have designed this study to provide a new guide for selecting drug targets.

## Methods

### Identification of drug target proteins

We used the DrugBank (version 3.0) [14] database to define drug target proteins. It contains nearly 6816 drug entries, including 760 FDA-approved drugs and 1822 of their targets, including 1661 proteins, 226 enzymes, 110 carriers, and 19 transporters. Using human UniProt/Swiss-Prot databases (release 2014.02) [10], 1578 non-redundant drug target proteins were defined and named as human drug target proteins or *hDP* ^+^. The remaining 17,575 human proteins were assigned to non-drug target proteins (named *hDP* ^0^).

To consider the possibility that the relevance of drug target protein properties may be over or underestimated depending on their gene family size, we built four datasets (A, B, C, and D). The first dataset A is composed of an initial 1,578 *hDP* ^+^ and 17,575 *hDP* ^0^. The second dataset B, derived from dataset A, contains only one representative protein from each gene family and thus has 792 *hDP* ^+^ and 8,361 *hDP* ^0^. For dataset C and D, if members of a gene family are derived from both *hDP* ^+^ and *hDP* ^0^, all genes in this gene family were excluded from the *hDP* ^0^ set. Thus, the third dataset C, derived from dataset A, has 1578 *hDP* ^+^ and 15,691 *hDP* ^0^, and the fourth dataset D, derived from dataset B, has 792 *hDP* ^+^ and 7949 *hDP* ^0^. In cases where a gene family has multiple members, the longest coding sequences (CDS) were selected to represent the gene family.

### Widely studied properties of drug target proteins

All properties (simple sequence properties, primary enzyme commission number, gene ontology terms, subcellular location, signal peptide cleavage, transmembrane helices, PEST regions that are rich in proline (P), glutamic acid (E), serine (S), and threonine (T), and secondary structure) tested in Bakheet and Doig [11], except for glycosylation, phosphorylation, and subcellular location, were reinvestigated for our four drug target datasets using the same bioinformatics tools and databases.

For more accurately and quantitatively analyzing post-translational protein modifications (PTMs), we used the PhosphoSitePlus database (March 4, 2014) [15], which is a manually curated collection of PTMs. It has collected nearly 212,556 PTM sites, and we used the top three PTMs for this study, including phosphorylation (160,338; 75.4%), ubiquitination (34,293; 16.1%), and acetylation (17,925; 8.4%).

Because the Swiss-Prot database has explained only about 18% of human proteins with respect to subcellular location, we used two additional subcellular localization databases: (1) manually curated LOCATE [16] database generated from a high-throughput immunofluorescence-based assay and peer-reviewed literature and (2) the comprehensively annotated Cell-PLoc [17] database using gene ontology, functional domain, and evolutionary conservation information. As a result, about 43% of human proteins had their subcellular location; however, the others still remain unrevealed. For these, we used five prediction programs (CELLO, pTarget, Proteome Analyst, WoLFPSORT, and MultiLoc) [18], and their subcellular locations were determined if they were supported by at least three prediction tools. In this study, we exploited ten subcellular location terms used in the LOCATE database as follows: cytoplasm, cytoskeleton, endoplasmic reticulum, extracellular, Golgi apparatus, lysosome, mitochondrion, nucleus, peroxisome, and plasma membrane.

### Newly proposed properties of drug target proteins

We downloaded the gene annotations for gene families through BioMart in the ENSEMBL database (release 75) [19], and the gene family was defined if it had at least two members.

Human essential and non-essential genes were obtained from Georgi et al. [20], who exploited genes with lethal and non-lethal phenotypes in the Mouse Genome Database. The dataset included 2472 essential genes and 3811 non-essential genes.

Gene expression data for 79 human tissues in U133A and GNF1H Affymetrix arrays were obtained from Su et al. [21]. We excluded all genes that were hit with other genes by a single probe. If multiple probe sets hit one gene, the probe set with the highest expression value was selected. The expression level (S) was defined by the average expression value in 79 tissues. The tissue specificity was calculated by

$$\tau =\frac{\sum_{j=1}^{n}\left (1-\frac{{log}_{2}S(j)}{{log}_{2}S_{max}} \right)}{n-1}, $$

where n (= 79) is the number of tissues and *S*(*j*) and *S*_*max*_ are gene expression level in tissue j and highest gene expression level within all tissues, respectively. Note that *S*(*j*) was set to 100 if it was less than 100 to minimize the influence of noise in the microarray data from the low expression level [22]. Higher *τ* value with ranges from 0 to 1 means a higher tissue specificity (i.e., greater variations in expression level across tissues).

SABLE [23] was used to predict the solvent accessibility of each amino acid in the protein sequences. The SABLE score ranged 0 to 99; values close to 0 indicate fully buried (i.e., solvent inaccessible) and close to 99 indicate fully exposed (i.e., solvent accessible). We used an average SABLE value for a protein as the solvent accessibility score.

### Statistical tests

To determine whether there was significantly different drug properties between *hDP* ^+^ and *hDP* ^0^, we performed two statistical tests: (1) a chi-square test and (2) a Wilcoxon rank-sum test for properties measured as discrete and continuous values, respectively.

### Predicting drug targets

We predicted drug targets by classifying proteins into two groups: *hDP* ^0^ proteins and *hDP* ^+^ proteins. For prediction, the properties of proteins were used as features for two machine learning approaches, SVM and RF, and R package (randomForest) and Liblinear were used for implementation [24]. Feature values were scaled into normalized values between 0 to 1 by calculating *X*=(*X*−*min*_*i*_)/(*max*_*i*_−*min*_*i*_), where *X* is the feature value and *min*_*i*_ and *max*_*i*_ are, respectively, the minimum and maximum values of the *i*th attribute. When we construct SVM and RF classifiers, we made the number of proteins in the two groups the same by reducing the number of proteins in *hDP* ^0^ with random selection. To construct the SVM classifier, the L2-regularized L2-loss support vector classification was used. The optimal error parameter (C) and radial bias parameter (*ε*) were set to 1.3 and 0.01, respectively. For SVM, we chose the parameter C with the “-C” option provided by Liblinear, which repeatedly selects the optimal value with training data [24]. Although the parameter C was recalculated during each cross-validation for all four data sets (A, B, C, and D), the same value was obtained. For the parameter *ε*, the default value was used. For RF, the size of the random subset of features evaluated at each node was calculated by *mtry*=*log*_2_(*number of features*+1), and the number of trees was set to 100. In general, with the more trees, the accuracy increases. However, the amount of improvements decreases when the number of trees becomes too large. Thus, the benefit of the prediction performance is less than the cost of the computation time to learn these additional trees [25].

We performed cross-validation to measure an accuracy of SVM and RF classifier based on widely used (*W*) and newly proposed (*N*) properties. In addition, we performed classification using statistically significant widely used (*W* ^′^) and newly proposed (*N* ^′^) features. Using only training data sets, we selected statistically significant features with p-value less than 0.05 at each cross-validation step. Recall, precision, and F-score were used as measurements: *recall*=*TP*/(*TP*+*FN*), *precision*=*TP*/(*TP*+*FP*), and *F*1=2×*recall*×*precision*/(*recall*+*precision*), where TP, FN, TN, and FP represent true positive (correctly predicted as *hDP* ^+^), false negative (incorrectly predicted as *hDP* ^0^), true negative (correctly predicted as *hDP* ^0^), and false positive (incorrectly predicted as *hDP* ^+^), respectively.

## Results and discussion

In this experiment, we essentially used the DrugBank database (version 3.0) and defined four different *hDP* ^+^ and *hDP* ^0^ datasets as described in the Materials and Methods and in Table 1. In comparison with Bakheet and Doig [11], who utilized 148 *hDP* ^+^ and 13,340 *hDP* ^0^ from the DrugBank database (version 1.0), our drug target protein datasets were significantly larger. Indeed, dataset D, which is the strictest for defining drug target proteins (see Materials and Methods for detail), has exhibited approximately five times higher *hDP* ^+^ (792 vs. 148) than Bakheet and Doig (2009) [11]. This indicates that our larger *hDP* ^+^ can have a higher statistical power, resulting in a higher sensitivity to slightly enriched and more specific properties of drug target proteins. Although all subsequent analyses were carried out for all four datasets (Additional file 1: Table S1), hereinafter we mainly present the results for dataset D. If there are inconsistent results among the four datasets, they are described in detail together with a discussion.

**Table 1:** Number of proteins for each dataset

	Set A	Set B	Set C	Set D
Number of *hDP* ^+^	1578	792	1578	792
Number of *hDP* ^0^	17,575	8361	15,691	7949

### Widely used properties of drug target proteins

**Fig. 2:**
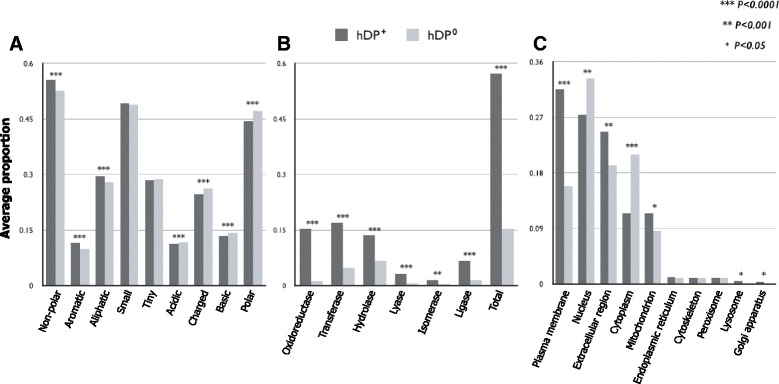
Analysis of widely used properties. The asterisk(*) represents the *p*-value of the statistical test. *One* asterisk means that the *p*-value is less than 0.05. *Two* asterisk means that the *p*-value is less than 0.001. *Three* asterisk means that the *p*-value is less than 0.0001. (**a**) Amino acid groups. (**b**) Primary enzyme class. (**c**) Subcellular location

We observed that *hDP* ^+^ tend to have more amino acids with hydrophobic side chains and less amino acids with electrically charged side chains than *hDP* ^0^, which is consistent with the study of Bakheet and Doig [11]. Non-polar, aromatic, or aliphatic amino acids prefer to be composed of *hDP* ^+^, whereas polar or charged (acidic, basic, and charged) amino acids are likely to be in *hDP* ^0^ (Fig. 2 a, Additional file 2: Figure S1). These observations were reconfirmed by using hydrophobicity, solubility, and the isoelectric point(pI). Namely, the average hydrophobicity score measured by hmoment [26] was significantly higher in *hDP* ^+^ than in *hDP* ^0^ (119.359 vs. 96.108, *P*=3.02×10^−12^), and from solubility of amino acid through improbability of expression in inclusion bodies, its median value of *hDP* ^+^ (0.703) was significantly lower than that of *hDP* ^0^ (0.703 vs. 0.733, *P*=9.08×10^−11^), confirming that *hDP* ^+^ are more hydrophobic and less polar than *hDP* ^0^. Further, the *hDP* ^0^’s pI was higher than that of *hDP* ^+^(7.457 vs. 7.128, *P*=9.63×10^−4^), supporting a preference for amino acids with charged side chains in *hDP* ^0^ (Table S1). It has been known that rapidly degraded proteins commonly contain PEST sequences [27]. We observed that *hDP* ^+^ have significantly less PEST sequences than *hDP* ^0^ (0.205 vs. 0.331, *P*=4.36×10^−13^), suggesting a longer lifetime of *hDP* ^+^. Between the two groups, there is no significant difference in the proportion of small (tiny) amino acids (Fig. 2 a). *hDP* ^+^ are longer than *hDP* ^0^ in average number of residues (418 vs. 342, *P*=1.14×10^−12^).

Because drug metabolism is closely related to enzymes [28], we checked and analyzed whether the *hDP* ^+^ when compared to *hDP* ^0^ contain relatively more enzyme proteins and which enzyme classes are dominant in *hDP* ^+^. As expected, more than half (453 out of 792, 57.1%) of *hDP* ^+^ are involved in enzyme activity, whereas 15.2% (1211 out of 7949) of *hDP* ^0^ are. All six enzyme classes have a significantly higher proportion of *hDP* ^+^ than in *hDP* ^0^ (Fig. 2 b, Additional file 3: Figure S2), which is inconsistent with Bakheet and Doig’s results. This inconsistency might have been caused by using distributions among only enzymes rather than using proportions of enzymes among all target proteins or non-target proteins.

We next investigated whether *hDP* ^+^ specifically include signal peptide sequences, which play an important role in the pharmacokinetics [29]. The frequency of signal peptide sequences in *hDP* ^+^ (347 out of 792) was significantly higher (0.452 vs. 0.226, *P*=1.49×10^−04^) than that in *hDP* ^0^ (1796 out of 7949), suggesting that *hDP* ^+^ are more likely to be secreted. Thus, we further explored which subcellular locations are preferentially associated with *hDP* ^+^. From the top five subcellular locations with a proportion > 10% in *hDP* ^+^, the plasma membrane, extracellular region, and mitochondrion were significantly favored as *hDP* ^+^ locations. In contrast, *hDP* ^0^ were frequently located in the nucleus and cytoplasm (Fig. 2 c, Additional file 4: Figure S3).

**Fig. 3:**
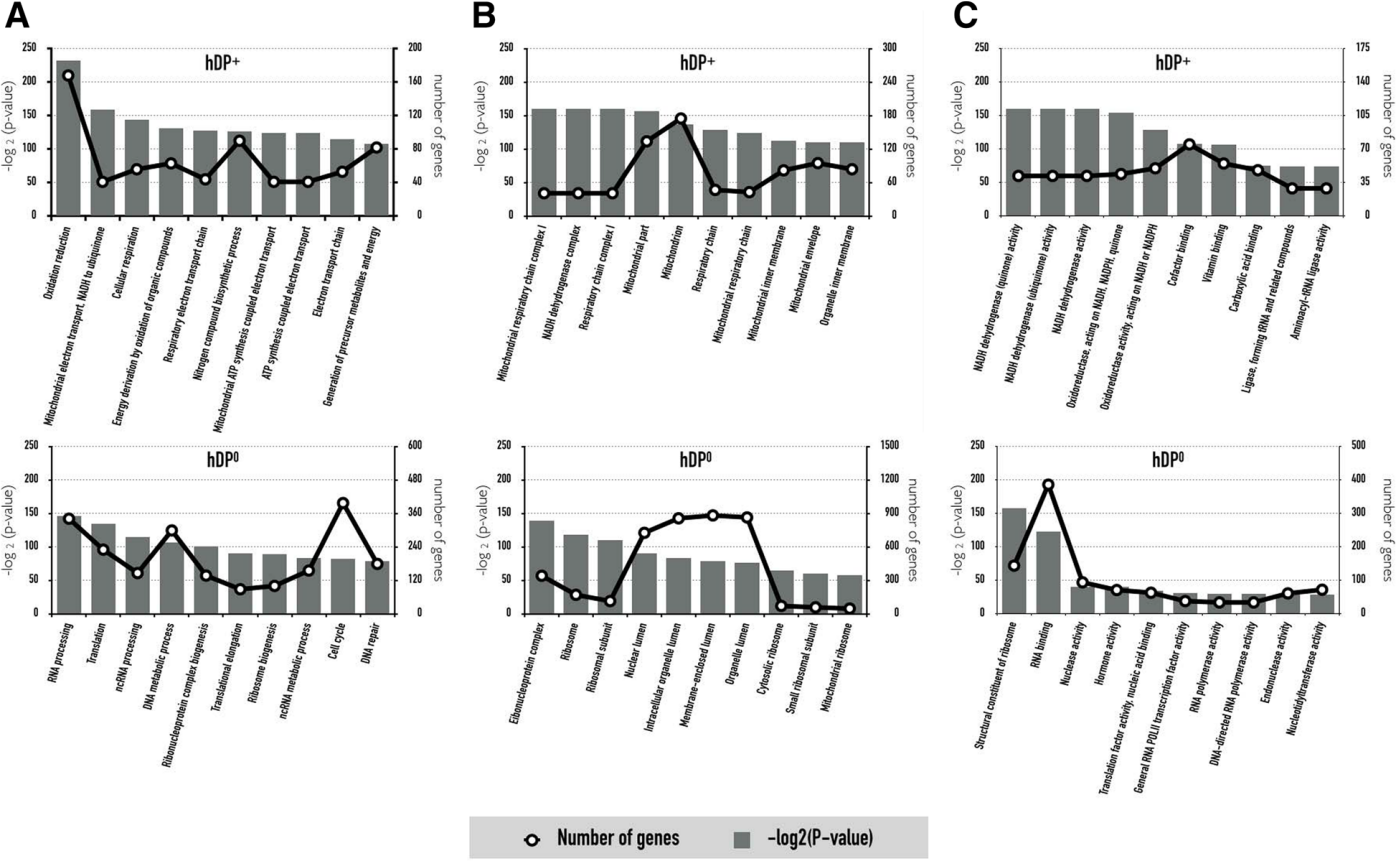
Analysis of gene ontology annotation. The *line graph* is the number of genes belonging to the corresponding GO term and the *bar graph* is taken from -log base 2 of the *p*-value calculated via DAVID. (**a**) Biological processes. (**b**) Cellular component. (**c**) Molecular function

From the analysis of gene ontology (GO) annotation^1^ [30] using the DAVID tool^2^ [31], we classified significantly enriched gene functional categories for *hDP* ^+^ and *hDP* ^0^. For the biological processes ontology, the significantly enriched gene categories for *hDP* ^+^ were oxidation reduction, mitochondrial electron transport, NADH to ubiquinone, cellular respiration, and energy derivation by oxidation of organic compounds, whereas RNA processing, translation, and DNA metabolic process were involved in *hDP* ^0^ (Fig. 3 a), indicating that drug target proteins are frequently involved in oxidation and reduction in cellular respiration. For the cellular component ontology, mitochondrion and membrane-related terms were enriched in *hDP* ^+^; however, *hDP* ^0^ had organelle favored categories including ribosome, nuclear, and intracellular (Fig. 3b), suggesting, consistent with the result of Bakheet and Doig [11], that drug target proteins favor mitochondrial membrane but not organelles. For the molecular function ontology, the gene categories favored for *hDP* ^+^ and *hDP* ^0^ included a set of NADH dehydrogenase activity, oxidoreductase activity, cofactor binding, vitamin binding, and carboxylic acid binding and a set of RNA binding, nuclease activity, hormone activity, translation factor activity, and RNA polymerase activity, respectively (Fig. 3 c), arguing that operational and informational genes [32] are preferentially involved in drug target and non-target proteins, respectively. The same analyses were performed by using the remaining three datasets (Additional file 5: Figure S4 for dataset A, Additional file 6: Figure S5 for dataset B, Additional file 7: Figure S6 for dataset C).

### Newly proposed properties of drug target proteins

**Fig. 4:**
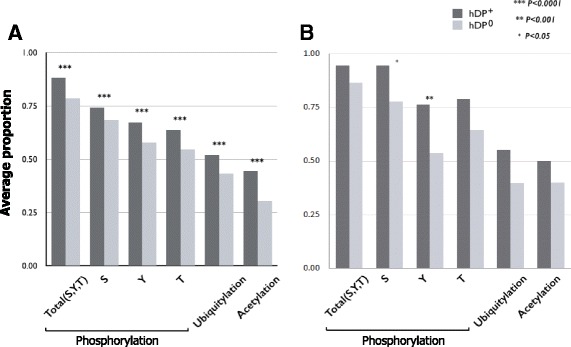
Analysis of PTMs. In phosphorylation, “S” indicate phosphorylation site in serin, “T” indicate threonine, and “Y” indicate tyrosine. The asterisk(*) represents the *p*-value of the statistical test. *One* asterisk means that the *p*-value is less than 0.05. *Two* asterisk means that the *p*-value is less than 0.001. *Three* asterisk means that the *p*-value is less than 0.0001. (**a**) Average proportion. (**b**) Average proportion in solvent accessible protein

PTMs play a central role in a wide range of cellular processes, including cellular activity, localization, differentiation, protein degradation, regulation and signaling, and interaction with other cellular molecules [33–36]. Folded proteins to attain their native state for proper biological function have distinct surface characteristics determining other molecules they interact with. Thus, to investigate whether proteins modified by major PTM types tend to be a target of drugs, we compared the proportions of proteins with PTMs between *hDP* ^+^ and *hDP* ^0^. Considering major PTM types, such as phosphorylation, ubiquitination, and acetylation [37, 38], *hDP* ^+^ contained relatively higher number of PTM residues than *hDP* ^0^. This type of pattern was likewise observed in all three PTM types (Fig. 4 a), which is inconsistent with Bakheet and Doig’s results. This inconsistency might have been caused by using computationally predicted PTM residues versus a manually curated collection of PTMs as used in this study. Because functional PTM residues are known to be enriched on the surface (i.e., solvent accessible) of folded proteins [23, 39, 40], we tested whether *hDP* ^+^ are more likely to hold potential functional PTM residues than *hDP* ^0^. We observed similar results (Fig. 4 b), confirming that proteins modified by major PTM types are more likely to be a target of drugs.

**Fig. 5:**
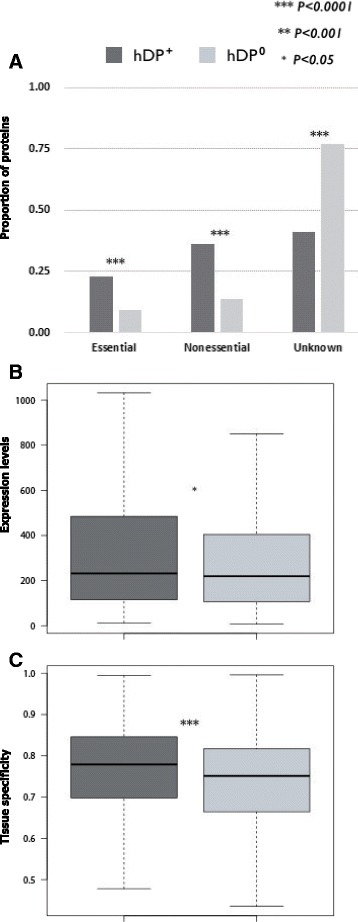
Analysis of newly proposed properties. The asterisk(*) represents the *p*-value of the statistical test. *One* asterisk means that the *p*-value is less than 0.05. *Two* asterisk means that the *p*-value is less than 0.001. *Three* asterisk means that the *p*-value is less than 0.0001. (**a**) Essential proteins. (**b**) Expression levels. (**c**) Tissue specificity

In general, drug target proteins have more interaction partner in protein-protein interaction network, and essential genes are enriched in protein complexes and tend to be highly expressed [20, 41, 42]. In this study, we addressed two issues. First, whether drug target proteins tend to be essential. Using predicted human essential proteins [20] (see methods in detail) it was shown that *hDP* ^+^ have more essential genes, but same pattern was also observed in non-essential genes (Fig. 5 a), indicating that as Yildirim et al. showed earlier [42], drug target proteins are not necessarily shown as higher essentials. Second, whether the gene expression level and tissue specificity influence the druggability of proteins. Using large-scale transcriptional profiling in 79 humans [21], it was revealed that *hDP* ^+^ have significantly higher expression level (Fig. 5 b) and greater tissue specificity (Fig. 5 c) than *hDP* ^0^.

### Predicting drug targets

We predicted drug targets using four datasets, A, B, C, and D, and the performance of the classification is shown in Table 2. Dataset C showed the best performance across all different combinations of features used. This may be because dataset C, which is derived from dataset A, excluded *hDP* ^0^ proteins that have the same gene family proteins in *hDP* ^+^. In addition, statistically significant features (*W* ^′^ + *N* ^′^) outperformed other features. Of all 75 features, 50 to 59 statistically significant features were chosen depending on training sets in the five-fold cross-validation (Additional file 8: Table S2), showing the importance of more relevant features for predicting drug targets. If a feature was not statistically significant in all cross-validation steps, it was indicated as “partially.” We also additionally performed 10-fold and 10x10-fold cross-validations, and the F-scores and standard derivations of 10-fold and 10x10-fold cross-validations are shown in Additional file 9: Figure S7. Although there were differences depending on the data sets, it is consistently shown that the best performances were obtained from the *W* ^′^ + *N* ^′^ feature of dataset C. Importantly, when newly proposed properties, like gene essentiality, gene expression levels, tissue specificity, and solvent accessibility, were incorporated, prediction performance increased, confirming the relevance of these features to the drug targets.

**Table 2:** Result for drug target protein prediction using machine learning methods

SVM	Recall	Precision	F1
Set A, *W*	0.7326	0.6594	0.6941
Set A, *W* ^′^	0.7516	0.7422	0.7469
Set A, *W*+*N*	0.7947	0.6681	0.7259
Set A, *W* ^′^ + *N* ^′^	0.8137	0.6982	0.7515
Set B, *W*	0.7866	0.6416	0.7067
Set B, *W* ^′^	0.7374	0.6496	0.6907
Set B, *W*+*N*	0.7424	0.6585	0.6979
Set B, *W* ^′^ + *N* ^′^	0.8018	0.6580	0.7228
Set C, *W*	0.7516	0.7808	0.7659
Set C, *W* ^′^	0.7972	0.8003	0.7987
Set C, *W*+*N*	0.8137	0.7965	0.8050
Set C, *W* ^′^ + *N* ^′^	**0.8409**	**0.8207**	**0.8307**
Set D, *W*	0.7820	0.7367	0.7587
Set D, *W* ^′^	0.8083	0.7588	0.7828
Set D, *W*+*N*	0.8120	0.7500	0.7798
Set D, *W* ^′^ + *N* ^′^	0.8271	0.7710	0.7981
RF			
Set A, *W*	0.7541	0.7682	0.7605
Set A, *W* ^′^	0.6483	0.8130	0.7260
Set A, *W*+*N*	0.7936	0.6763	0.7299
Set A, *W* ^′^ + *N* ^′^	0.8229	0.6986	0.7556
Set B, *W*	0.7821	0.6547	0.7124
Set B, *W* ^′^	0.7490	0.6493	0.6953
Set B, *W*+*N*	0.7551	0.7805	0.7677
Set B, *W* ^′^ + *N* ^′^	0.8076	0.6767	0.7363
Set C, *W*	0.7847	0.7358	0.7589
Set C, *W* ^′^	0.8165	0.7960	0.8057
Set C, *W*+*N*	0.8292	0.8118	0.8200
Set C, *W* ^′^ + *N* ^′^	**0.8509**	**0.8218**	**0.8354**
Set D, *W*	0.7885	0.7409	0.7636
Set D, *W* ^′^	0.8343	0.7564	0.7934
Set D, *W*+*N*	0.8305	0.7550	0.7908
Set D, *W* ^′^ + *N* ^′^	0.8382	0.7818	0.8088

Feature sets *W* and *N* represent widely used and newly proposed properties, respectively. *W* ^′^ and *N* ^′^ represent statistically significant widely used and newly proposed properties, respectively

The underline bold numbers indicate the highest values in each evaluation

Bull and Doig [12] and Huang et al. [13] also predicted drug targets. Bull and Doig [12] employed the RF method with extended protein properties from Bakheet and Doig [11], and Huang et al. [13] used the SVM method with the same protein properties as those in Bakheet and Doig [11]. The accuracy of Bull and Doig [12] and Huang et al. [13] were an F-score of 0.8237 and a G-mean of 0.7813, respectively. Because datasets used in Bull and Doig [12], Huang et al. [13], and this study were somewhat different due to different versions of DrugBank, it is hard to directly compare their results with ours. However, the accuracy values of the F-score of our approach incorporating newly proposed properties were higher than those from the previous two approaches. In addition, because the approach in Huang et al. [13] was similar to that of our study using dataset A with features of *W* ^′^, we can infer that dataset C with features of *W* ^′^ + *N* ^′^ outperforms the approach in Huang et al. [13].

## Conclusions

In this study, we proposed new properties (essentiality, expression pattern, PTMs, and solvent accessibility) for effectively identifying drug target proteins. To this end, we performed a highly controlled experimental study (in silico) in order to minimize statistical biases due to involvement of redundant duplicated genes. Although it has been known that essential proteins are indispensable to the viability of an organism and the loss of just one of them is sufficient to lead to lethality or infertility [41, 42], intriguingly we observed drug targetability and protein essentiality are decoupled. We also revealed that druggability of proteins has high expression level and tissue specificity. To investigate whether drug target proteins appear to be PTMs, as different from previous studies [11, 12], we used a manually curated large collection of PTMs with protein structure information. Using three major types of PTM (phosphorylation, acetylation, and ubiquitination), functional PTM residues are enriched in drug target proteins. We also reassessed the widely used properties of drug target proteins. Using more comprehensive and refined set of protein properties with more powerful methodologies, we confirmed and extended that drug target proteins (1) are likely to have more hydrophobic, less polar, less PEST sequences, no preference in the proportion of small amino acids, more increase in length of residues, and more signal peptide sequences higher and (2) are more involved in enzyme catalysis, oxidation and reduction in cellular respiration, and operational genes. To build a classifier distinguishing between drug and non-drug target proteins, we utilized both newly proposed properties and widely used properties and we achieved much higher accuracy rate compared to that using existing the widely used properties. As a result, we expect that our new properties as well as extended existing ones will help to infer drug-target interactions more reliably.

## Endnotes

^1^ http://geneontology.org

^2^ http://david.abcc.ncifcrf.gov

## Additional files

Additional file 1 **Table S1.** Proportion and average as binary and continuous and p-value for all subsets. (PDF 232 kb)

Additional file 2 **Figure S1.** Result for amino acid group: (A) for set A. (B) for set B. (C) for Set C. (PDF 147 kb)

Additional file 3 **Figure S2.** Result for primary enzyme class: (A) for set A. (B) for set B. (C) for Set C. (PDF 146 kb)

Additional file 4 **Figure S3.** Result of subcellular location: (A) for set A. (B) for set B. (C) for Set C. (PDF 160 kb)

Additional file 5 **Figure S4.** Result of gene ontology annotation for set A: (A) Biological processes. (B) Cellular component. (C) Molecular function. (PDF 306 kb)

Additional file 6 **Figure S5.** Result of gene ontology annotation for set B: (A) Biological processes. (B) Cellular component. (C) Molecular function. (PDF 456 kb)

Additional file 7 **Figure S6.** Result of gene ontology annotation for set C: (A) Biological processes. (B) Cellular component. (C) Molecular function. (PDF 509 kb)

Additional file 8 **Table S2.** Statistically significant widely used (W’) and newly proposed (N’) features. (PDF 180 kb)

Additional file 9 **Figure S7.** 10-fold and 10x10-fold cross-validations result in terms of the F-score and the standard derivation. (A) 10-fold cross-validation for SVM. (B) 10-fold cross-validation for RF. (C) 10X10 fold cross-validation for SVM. (D) 10X10 fold cross-validation for RF. (PDF 207 kb)
