# Supplementary material for: In silico re-identification of properties of drug target proteins
Source: BMC Bioinformatics. 2017 May 31;18(Suppl 7):248. doi: 10.1186/s12859-017-1639-3 (PMC5471946; doi:10.1186/s12859-017-1639-3)
Supplement: Supplementary file 1 — Table S1. Proportion and average as binary and continuous and p-value for all subsets. (PDF 232 kb) [file 12859_2017_1639_MOESM1_ESM.pdf]

Supplementary Table 1. Proportion and average as binary and continuous and p-value for all subsets.

Class 1	Class 2	t-test	p-value				Set A		Set B		Set C		Set D	
			Set A	Set B	Set C	Set D	hDP^+	hDP^0	hDP^+	hDP^0	hDP^+	hDP^0	hDP^+	hDP^0
Simple properties	Hydrophobicity	ranksum	7.57E-10	3.02E-12	2.22E-12	3.02E-12	134.671	118.484	119.359	97.028	134.671	116.020	119.359	96.108
Simple properties	Residues	ranksum	1.55E-09	9.36E-12	2.42E-12	1.14E-12	478.000	421.000	418.000	346.000	478.000	413.000	418.000	342.000
Simple properties	Isoelectric point	ranksum	6.42E-02	1.05E-03	3.40E-02	9.64E-04	7.274	7.441	7.128	7.458	7.274	7.455	7.128	7.458
Simple properties	Improbability	ranksum	5.65E-14	1.94E-10	2.17E-15	9.08E-11	0.719	0.742	0.703	0.732	0.719	0.744	0.703	0.733
Simple properties	Ala	ranksum	4.77E-03	2.62E-02	5.77E-03	2.12E-02	7.053	6.818	7.299	6.964	7.053	6.823	7.299	6.960
Simple properties	Cys	ranksum	8.07E-01	9.90E-01	7.11E-01	8.01E-01	2.023	2.030	1.975	1.968	2.023	2.013	1.975	1.955
Simple properties	Asp	ranksum	1.20E-04	1.47E-04	1.19E-04	1.53E-04	4.771	4.569	4.841	4.566	4.771	4.569	4.841	4.569
Simple properties	Glu	ranksum	8.59E-29	3.39E-11	1.36E-35	4.87E-13	5.985	6.655	6.280	6.818	5.985	6.757	6.280	6.897
Simple properties	Phe	ranksum	4.15E-96	5.61E-35	2.15E-108	7.83E-39	4.467	3.575	4.240	3.497	4.467	3.513	4.240	3.448
Simple properties	Gly	ranksum	2.57E-17	3.75E-17	8.24E-22	8.01E-19	6.859	6.298	6.910	6.140	6.859	6.219	6.910	6.087
Simple properties	His	ranksum	2.79E-08	8.39E-01	9.96E-09	8.28E-01	2.316	2.448	2.431	2.400	2.316	2.452	2.431	2.400
Simple properties	Ile	ranksum	9.23E-74	7.98E-36	6.35E-84	1.09E-38	5.196	4.188	5.101	4.124	5.196	4.103	5.101	4.072
Simple properties	Lys	ranksum	7.30E-03	2.79E-01	2.58E-04	1.41E-01	5.395	5.442	5.593	5.612	5.395	5.514	5.593	5.645
Simple properties	Leu	ranksum	2.53E-06	7.32E-01	2.30E-07	9.09E-01	10.220	9.950	10.166	10.294	10.220	9.913	10.166	10.274
Simple properties	Met	ranksum	1.19E-37	6.18E-12	1.38E-39	2.49E-12	2.465	2.105	2.414	2.128	2.465	2.093	2.414	2.120
Simple properties	Asn	ranksum	7.71E-14	1.18E-10	8.94E-16	5.09E-11	3.651	3.403	3.571	3.220	3.651	3.378	3.571	3.215
Simple properties	Pro	ranksum	1.42E-12	2.37E-07	4.88E-14	8.45E-08	5.208	5.574	5.172	5.556	5.208	5.616	5.172	5.581
Simple properties	Gln	ranksum	7.37E-72	2.42E-30	2.41E-81	2.24E-32	3.750	4.457	3.891	4.545	3.750	4.527	3.891	4.580
Simple properties	Arg	ranksum	4.99E-23	2.00E-17	3.90E-26	1.58E-18	5.137	5.556	5.070	5.641	5.137	5.584	5.070	5.668
Simple properties	Ser	ranksum	1.53E-61	1.28E-31	6.31E-67	4.61E-34	6.875	7.743	6.606	7.574	6.875	7.801	6.606	7.632
Simple properties	Thr	ranksum	7.31E-06	1.18E-04	9.40E-08	4.82E-05	5.186	5.057	5.100	4.936	5.186	5.031	5.100	4.926
Simple properties	Val	ranksum	2.92E-77	1.56E-34	1.50E-89	8.73E-38	6.831	5.876	6.777	5.858	6.831	5.790	6.777	5.817
Simple properties	Trp	ranksum	5.86E-37	2.51E-07	1.53E-45	1.70E-08	1.433	1.111	1.313	1.136	1.433	1.067	1.313	1.124

Simple properties	Tyr	ranksum	9.61E-53	2.94E-37	7.19E-62	2.80E-40	3.149	2.632	3.112	2.451	3.149	2.569	3.112	2.427
Simple properties	Tiny	ranksum	1.41E-05	3.51E-01	9.20E-05	3.95E-01	28.571	29.167	28.517	28.627	28.571	29.123	28.517	28.634
Simple properties	Small	ranksum	7.55E-01	1.50E-01	7.58E-01	1.19E-01	49.402	49.442	49.193	48.918	49.402	49.356	49.193	48.905
Simple properties	Aliphatic	ranksum	2.64E-59	5.59E-23	6.26E-67	1.52E-25	29.550	27.603	29.597	27.907	29.550	27.421	29.597	27.807
Simple properties	Aromatic	ranksum	2.44E-68	1.19E-34	2.09E-79	1.54E-38	11.724	10.236	11.450	9.906	11.724	10.076	11.450	9.813
Simple properties	Non_polar	ranksum	3.27E-94	1.95E-38	4.17E-111	1.82E-43	55.837	52.889	55.510	52.941	55.837	52.533	55.510	52.716
Simple properties	Polar	ranksum	3.29E-94	1.96E-38	4.20E-111	1.84E-43	44.164	47.111	44.490	47.059	44.164	47.467	44.490	47.284
Simple properties	Charged	ranksum	1.82E-39	7.28E-15	1.61E-49	2.82E-17	24.144	25.769	24.807	26.073	24.144	26.034	24.807	26.216
Simple properties	Basic	ranksum	7.55E-37	3.67E-16	2.09E-45	2.42E-18	13.110	13.934	13.386	14.130	13.110	14.074	13.386	14.213
Simple properties	Acidic	ranksum	7.07E-13	3.22E-04	2.73E-16	4.62E-05	10.813	11.424	11.260	11.561	10.813	11.517	11.260	11.635
EC	1. Oxidoreductases	chi-squire	2.20E-16	2.20E-16	2.20E-16	2.20E-16	0.295	0.090	0.269	0.109	0.295	0.067	0.269	0.085
EC	2. Transferases	chi-squire	2.20E-16	2.20E-16	2.20E-16	2.20E-16	0.307	0.355	0.296	0.320	0.307	0.356	0.296	0.320
EC	3. Hydrolases	chi-squire	4.70E-11	1.69E-11	2.95E-16	5.62E-13	0.239	0.409	0.238	0.418	0.239	0.413	0.238	0.434
EC	4. Lyases	chi-squire	2.20E-16	3.85E-13	2.20E-16	3.18E-14	0.057	0.025	0.055	0.037	0.057	0.024	0.055	0.037
EC	5. Isomerases	chi-squire	2.20E-03	7.02E-03	1.92E-04	4.02E-03	0.022	0.028	0.024	0.032	0.022	0.026	0.024	0.033
EC	6. Ligases	chi-squire	2.49E-10	2.20E-16	2.99E-09	2.20E-16	0.079	0.094	0.117	0.084	0.079	0.113	0.117	0.091
EC	Total	chi-squire	2.20E-16	2.20E-16	2.20E-16	2.20E-16	0.511	0.184	0.572	0.166	0.511	0.160	0.572	0.152
Subcellular location	Cytoplasm	chi-squire	2.20E-16	7.46E-14	2.20E-16	2.04E-10	0.110	0.220	0.115	0.231	0.110	0.251	0.115	0.211
Subcellular location	Cytoskeleton	chi-squire	9.67E-01	1.00E+00	4.43E-01	1.00E+00	0.010	0.010	0.010	0.010	0.010	0.012	0.010	0.010
Subcellular location	Endoplasmic reticulum	chi-squire	9.67E-01	7.62E-01	5.48E-01	8.72E-01	0.010	0.010	0.011	0.010	0.010	0.012	0.011	0.010
Subcellular location	Extracellular region	chi-squire	1.00E+00	2.17E-06	2.59E-01	4.01E-04	0.180	0.180	0.246	0.177	0.180	0.168	0.246	0.193
Subcellular location	Golgi apparatus	chi-squire	7.67E-02	1.00E-03	5.20E-01	1.40E-02	0.001	0.000	0.004	0.000	0.001	0.001	0.004	0.001
Subcellular location	Lysosome	chi-squire	2.03E-02	1.90E-02	1.02E-01	1.52E-02	0.003	0.001	0.005	0.001	0.003	0.001	0.005	0.001
Subcellular location	Mitochondrion	chi-squire	1.08E-02	1.31E-02	5.79E-03	1.16E-02	0.110	0.090	0.115	0.088	0.110	0.088	0.115	0.087
Subcellular location	Nucleus	chi-squire	9.88E-02	1.31E-02	1.52E-01	7.18E-04	0.290	0.310	0.274	0.327	0.290	0.307	0.274	0.334
Subcellular location	Peroxisome	chi-squire	9.67E-01	1.00E+00	9.93E-01	1.00E+00	0.010	0.010	0.010	0.011	0.010	0.010	0.010	0.010

Subcellular location	Plasma membrane	chi-squire	2.20E-16	2.20E-16	2.20E-16	2.20E-16	0.279	0.170	0.316	0.170	0.279	0.150	0.316	0.158
PEST region	PEST region	chi-squire	2.20E-16	3.25E-12	2.20E-16	4.36E-13	25.475	36.558	0.205	0.326	0.583	0.672	0.205	0.331
Secondary structure	H(alpha-helix)	ranksum	2.31E-01	8.42E-02	2.58E-02	3.73E-02	35.600	35.600	37.400	37.900	35.600	35.900	37.400	38.100
Secondary structure	E(beta-chain)	ranksum	2.73E-64	5.01E-28	4.89E-77	1.42E-31	25.500	22.000	24.800	21.800	25.500	21.600	24.800	21.600
Secondary structure	T(turn)	ranksum	2.11E-07	3.37E-03	8.40E-07	3.79E-03	21.700	22.400	21.200	21.900	21.700	22.400	21.200	21.900
Secondary structure	C(coil)	ranksum	2.60E-19	5.26E-08	4.11E-21	9.38E-09	19.900	21.200	19.800	21.100	19.900	21.300	19.800	21.200
Signal peptide cleavage	SignalP	chi-squire	2.20E-16	9.01E-15	2.20E-16	2.20E-16	0.276	0.157	0.244	0.140	0.276	0.139	0.244	0.134
Transmembrane helices	TMHMM	chi-squire	2.20E-16	2.20E-16	2.20E-16	2.20E-16	0.444	0.247	0.345	0.213	0.444	0.225	0.345	0.205
Transmembrane helices	TMHMM-7helices	chi-squire	2.22E-12	8.39E-09	2.20E-16	3.03E-11	0.069	0.034	0.042	0.014	0.069	0.028	0.042	0.012
PTMs	Phosphorylation	chi-squire	1.90E-14	2.20E-16	8.48E-15	1.24E-10	0.898	0.822	0.883	0.659	0.898	0.820	0.883	0.785
PTMs	Phosphorylation-S	chi-squire	2.82E-06	2.20E-16	3.71E-06	2.65E-05	0.742	0.685	0.723	0.466	0.742	0.685	0.723	0.648
PTMs	Phosphorylation-Y	chi-squire	4.45E-06	2.20E-16	4.05E-06	7.69E-06	0.638	0.578	0.609	0.317	0.638	0.577	0.609	0.525
PTMs	Phosphorylation-T	chi-squire	2.20E-16	2.20E-16	2.20E-16	2.20E-16	0.672	0.548	0.658	0.288	0.672	0.543	0.658	0.493
PTMs	Ubiquitination	chi-squire	5.17E-03	6.35E-07	3.75E-02	2.56E-06	0.443	0.406	0.520	0.428	0.443	0.416	0.520	0.433
PTMs	Acetylation	chi-squire	3.04E-13	4.08E-16	3.39E-12	2.20E-15	0.411	0.320	0.444	0.303	0.411	0.324	0.444	0.306
Essentiality	Essential	chi-squire	2.20E-16	2.20E-16	2.20E-16	2.20E-16	0.236	0.120	0.227	0.093	0.235	0.116	0.227	0.091
Gene expression pattern	Expression level	ranksum	4.50E-04	8.22E-04	9.34E-04	1.55E-03	212.706	201.868	219.588	217.907	212.706	203.091	219.588	231.931
Gene expression pattern	Tissue specificity	ranksum	2.20E-16	2.81E-16	2.20E-16	2.20E-16	0.788	0.762	0.779	0.753	0.788	0.758	0.779	0.751
Protein duplication	PD>=2	chi-squire	1.80E-11	8.39E-09	2.20E-16	4.84E-14	0.827	0.707	0.489	0.385	0.827	0.672	0.489	0.353
Protein surface accessibility	SABLE	ranksum	2.20E-16	2.20E-16	2.20E-16	2.20E-16	22.834	26.803	22.938	27.593	22.834	27.222	22.938	27.868
Protein surface accessibility	SABLE&phospolylation	chi-squire	3.73E-01	2.24E-01	3.54E-01	2.20E-01	0.943	0.896	0.947	0.867	0.943	0.895	0.947	0.866
Protein surface accessibility	SABLE&phospolylation(s)	chi-squire	6.41E-02	2.26E-02	5.80E-02	2.20E-02	0.925	0.816	0.947	0.780	0.925	0.813	0.947	0.779
Protein surface accessibility	SABLE&phospolylation(T)	chi-squire	7.78E-02	1.01E-01	7.25E-02	9.70E-02	0.811	0.689	0.789	0.648	0.811	0.687	0.789	0.647
Protein surface accessibility	SABLE&phospolylation(Y)	chi-squire	5.92E-02	9.81E-03	5.16E-02	9.61E-03	0.736	0.599	0.763	0.539	0.736	0.594	0.763	0.539
Protein surface accessibility	SABLE&Acetylation	chi-squire	4.44E-01	2.86E-01	4.28E-01	2.85E-01	0.528	0.466	0.500	0.401	0.528	0.464	0.500	0.401
Protein surface accessibility	SABLE&ubiqitination	chi-squire	5.16E-02	8.49E-02	4.68E-02	8.15E-02	0.566	0.423	0.553	0.401	0.566	0.421	0.553	0.400
