# Supplementary figures and images for: In silico re-identification of properties of drug target proteins
Source: BMC Bioinformatics. 2017 May 31;18(Suppl 7):248. doi: 10.1186/s12859-017-1639-3 (PMC5471946; doi:10.1186/s12859-017-1639-3)

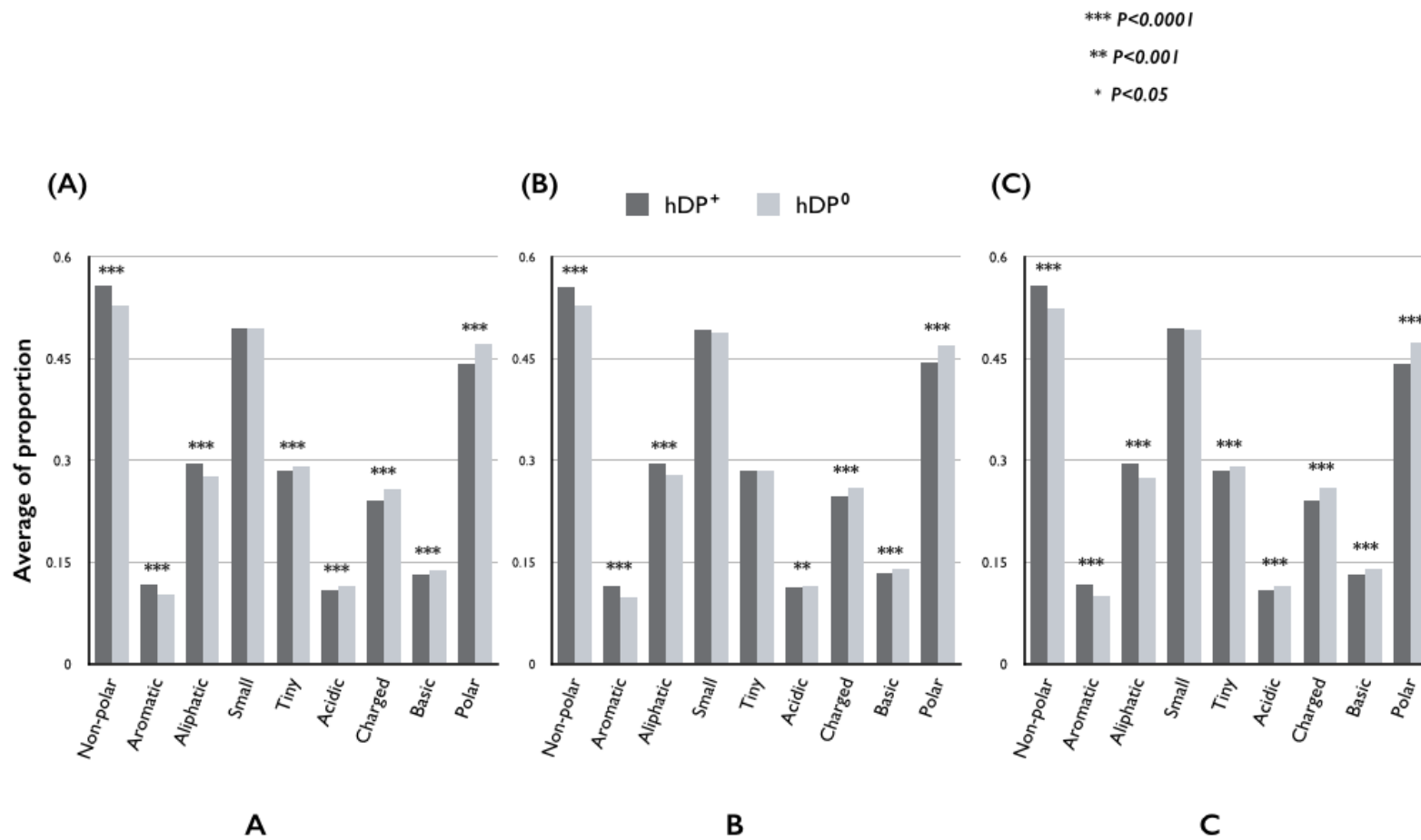

Supplementary Figure 1. Result for amino acid group: (A) for set A. (B) for set B. (C) for Set C.

Supplement: Supplementary file 2 — Figure S1. Result for amino acid group: (A) for set A. (B) for set B. (C) for Set C. (PDF 147 kb) [file 12859_2017_1639_MOESM2_ESM.pdf]

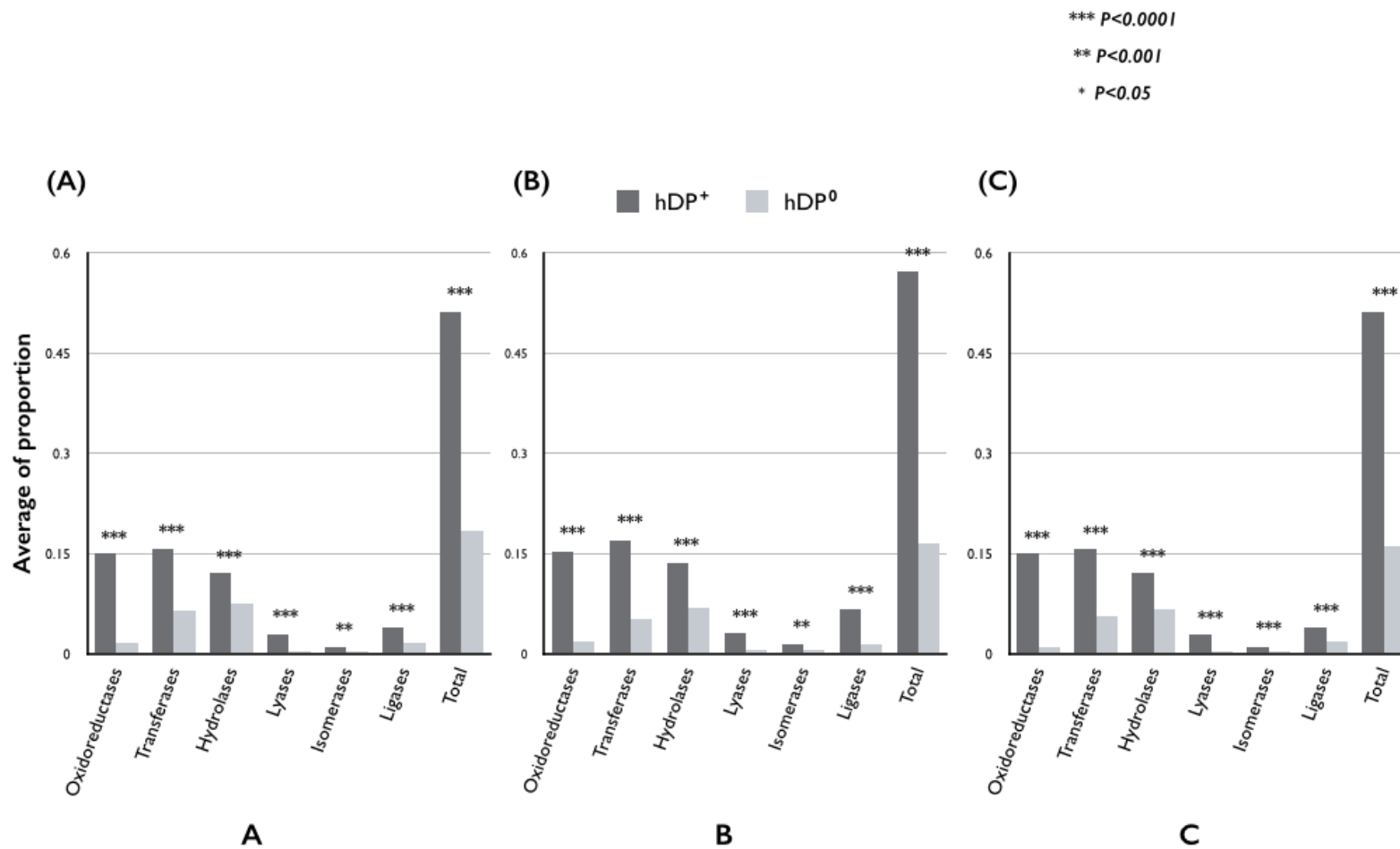

Supplementary Figure 2. Result for primary enzyme class: (A) for set A. (B) for set B. (C) for Set C.

Supplement: Supplementary file 3 — Figure S2. Result for primary enzyme class: (A) for set A. (B) for set B. (C) for Set C. (PDF 146 kb) [file 12859_2017_1639_MOESM3_ESM.pdf]

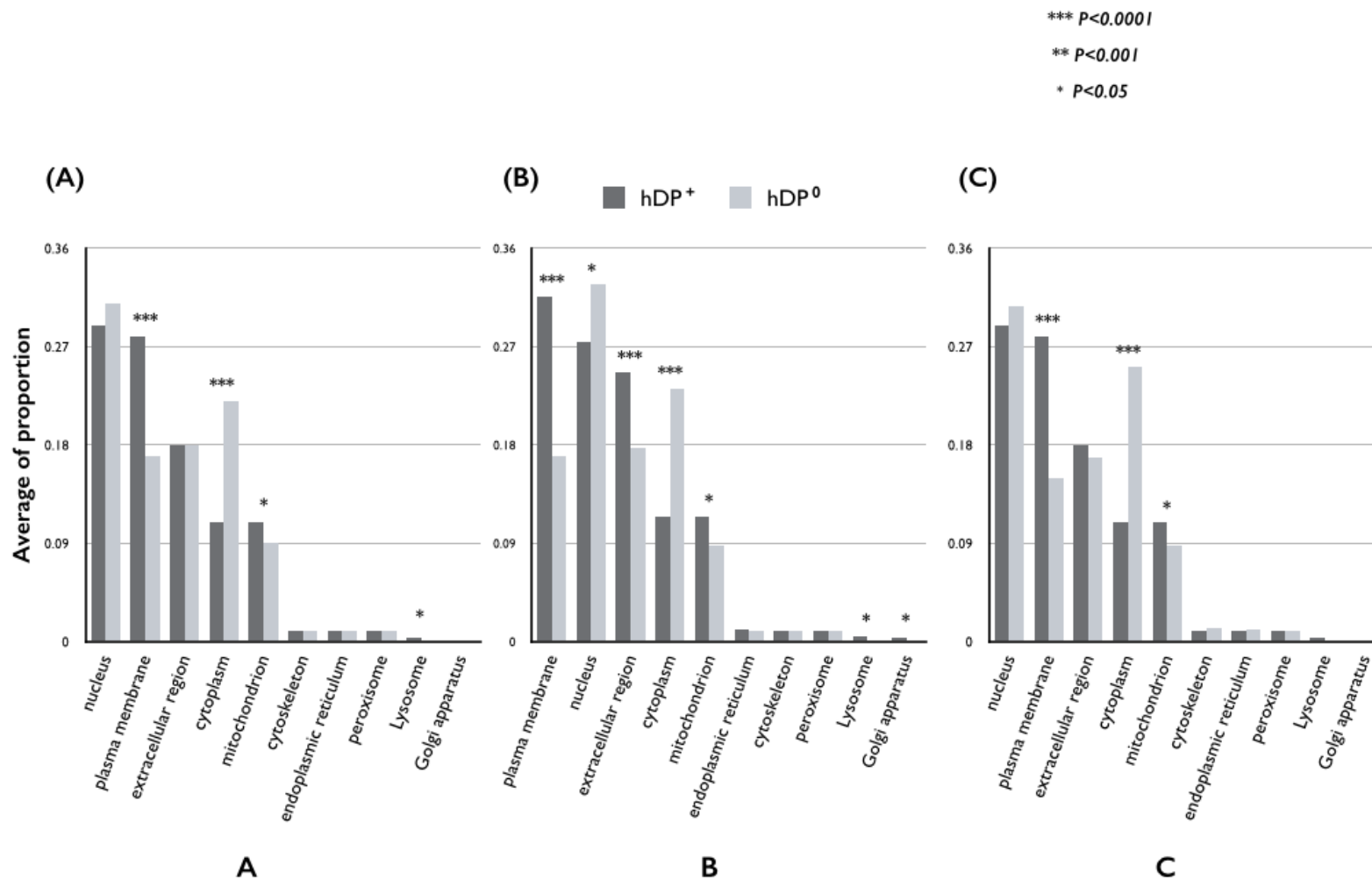

Supplementary Figure 3. Result of subcellular location: (A) for set A. (B) for set B. (C) for Set C.

Supplement: Supplementary file 4 — Figure S3. Result of subcellular location: (A) for set A. (B) for set B. (C) for Set C. (PDF 160 kb) [file 12859_2017_1639_MOESM4_ESM.pdf]
