# Supplementary material for: In silico re-identification of properties of drug target proteins
Source: BMC Bioinformatics. 2017 May 31;18(Suppl 7):248. doi: 10.1186/s12859-017-1639-3 (PMC5471946; doi:10.1186/s12859-017-1639-3)
Supplement: Supplementary file 7 — Figure S6. Result of gene ontology annotation for set C: (A) Biological processes. (B) Cellular component. (C) Molecular function. (PDF 509 kb) [file 12859_2017_1639_MOESM7_ESM.pdf]

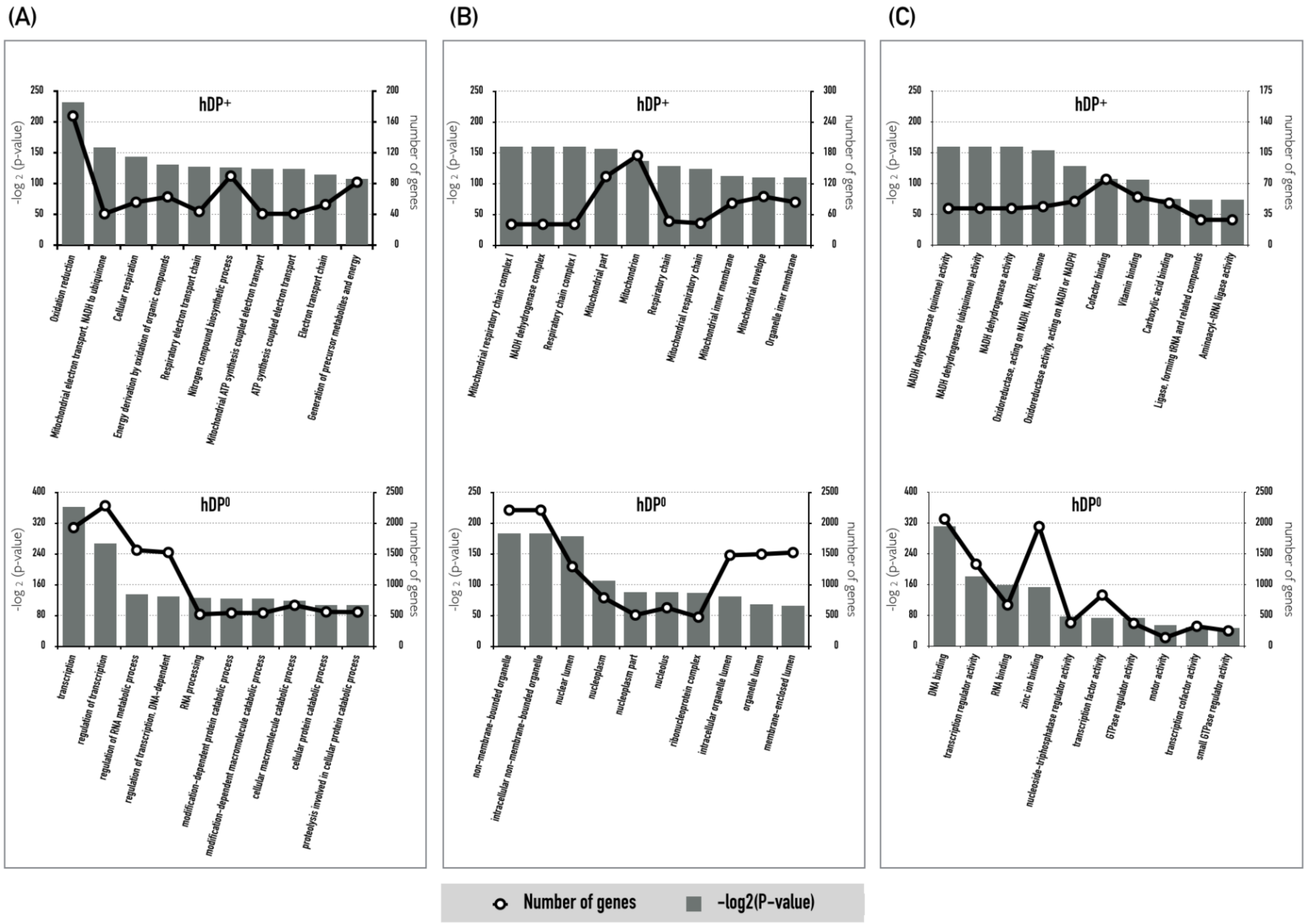

Supplementary Figure 6. Result of gene ontology annotation for set C: (A) Biological processes. (B) Cellular component. (C) Molecular function.
