# Supplementary material for: In silico re-identification of properties of drug target proteins
Source: BMC Bioinformatics. 2017 May 31;18(Suppl 7):248. doi: 10.1186/s12859-017-1639-3 (PMC5471946; doi:10.1186/s12859-017-1639-3)
Supplement: Supplementary file 8 — Table S2. Statistically significant widely used (W’) and newly proposed (N’) features. (PDF 180 kb) [file 12859_2017_1639_MOESM8_ESM.pdf]

Supplementary Table 2. Statistically significant widely used (W') and newly proposed (N') features.

Widely used properties/ Newly proposed properties	Class 2	Features for random forest				Features for support vector machine			
		Set A	Set B	Set C	Set D	Set A	Set B	Set C	Set D
Widely used properties	Hydrophobicity	W'	W'	W'	W'	W'	W'	W'	W'
Widely used properties	Residues	W'	W'	W'	W'	W'	W'	W'	W'
Widely used properties	Isoelectric point	x	W'	W'	W'	x	x	x	x
Widely used properties	Improbability	W'	W'	W'	W'	W'	W'	W'	W'
Widely used properties	Ala	W'(partially)	x	W'	x	x	x	x	x
Widely used properties	Cys	x	x	x	x	x	x	x	x
Widely used properties	Asp	W'	W'	W'	W'	W'	W'	W'	W'
Widely used properties	Glu	W'	W'	W'	W'	W'	W'	W'	W'
Widely used properties	Phe	W'	W'	W'	W'	W'	W'	W'	W'
Widely used properties	Gly	W'	W'	W'	W'	W'	W'	W'	W'
Widely used properties	His	W'	x	W'	x	W'	x	W'	x
Widely used properties	Ile	W'	W'	W'	W'	W'	W'	W'	W'
Widely used properties	Lys	W'(partially)	x	W'	x	x	x	W'	x
Widely used properties	Leu	W'	x	W'	x	W'	x	W'	x
Widely used properties	Met	W'	W'	W'	W'	W'	W'	W'	W'
Widely used properties	Asn	W'	W'	W'	W'	W'	W'	W'	W'
Widely used properties	Pro	W'	W'	W'	W'	W'	W'	W'	W'
Widely used properties	Gln	W'	W'	W'	W'	W'	W'	W'	W'
Widely used properties	Arg	W'	W'	W'	W'	W'	W'	W'	W'
Widely used properties	Ser	W'	W'	W'	W'	W'	W'	W'	W'
Widely used properties	Thr	W'	W'	W'	W'	W'	W'	W'	W'
Widely used properties	Val	W'	W'	W'	W'	W'	W'	W'	W'
Widely used properties	Trp	W'	W'	W'	W'	W'	W'	W'	W'

Widely used properties	Tyr	W'	W'	W'	W'	W'	W'	W'	W'
Widely used properties	Tiny	W'	x	W'	x	W'	x	W'	x
Widely used properties	Small	x	x	x	x	x	x	x	x
Widely used properties	Aliphatic	W'	W'	W'	W'	W'	W'	W'	W'
Widely used properties	Aromatic	W'	W'	W'	W'	W'	W'	W'	W'
Widely used properties	Non_polar	W'	W'	W'	W'	W'	W'	W'	W'
Widely used properties	Polar	W'	W'	W'	W'	W'	W'	W'	W'
Widely used properties	Charged	W'	W'	W'	W'	W'	W'	W'	W'
Widely used properties	Basic	W'	W'	W'	W'	W'	W'	W'	W'
Widely used properties	Acidic	W'	W'	W'	W'	W'	W'	W'	W'
Widely used properties	1. Oxidoreductases	W'	W'	W'	W'	W'	W'	W'	W'
Widely used properties	2. Transferases	W'	W'	W'	W'	W'	W'	W'	W'
Widely used properties	3. Hydrolases	W'	W'	W'	W'	W'	W'	W'	W'
Widely used properties	4. Lyases	W'	W'	W'	W'	W'	W'	W'	W'
Widely used properties	5. Isomerases	W'	W'	W'	W'	x	x	W'	x
Widely used properties	6. Ligases	W'	W'	W'	W'	W'	W'	W'	W'
Widely used properties	EC(Total)	W'	W'	W'	W'	W'	W'	W'	W'
Widely used properties	Cytoplasm	W'	W'	W'	W'	W'	W'	W'	W'
Widely used properties	Cytoskeleton	x	x	x	x	x	x	x	x
Widely used properties	Endoplasmic reticulum	x	x	x	x	x	x	x	x
Widely used properties	Extracellular region	x	W'	x	W'	x	W'	x	W'
Widely used properties	Golgi apparatus	x	W'	x	W'	x	x	x	x
Widely used properties	Lysosome	W'(partially)	W'	x	W'	x	x	x	x
Widely used properties	Mitochondrion	W'	W'	W'	W'	x	x	x	x
Widely used properties	Nucleus	x	W'	x	W'	x	x	x	x
Widely used properties	Peroxisome	x	x	x	x	x	x	x	x

Widely used properties	Plasma membrane	W'	W'	W'	W'	W'	W'	W'	W'
Widely used properties	PEST region	W'	W'	W'	W'	W'	W'	W'	W'
Widely used properties	H(alpha-helix)	x	x	W'(partially)	W'(partially)	x	x	x	x
Widely used properties	E(beta-chain)	W'	W'	W'	W'	W'	W'	W'	W'
Widely used properties	T(turn)	W'	W'	W'	W'	W'(partially)	x	W'(partially)	x
Widely used properties	C(coil)	W'	W'	W'	W'	W'	W'	W'	W'
Widely used properties	SignalP	W'	W'	W'	W'	W'	W'	W'	W'
Widely used properties	TMHMM	W'	W'	W'	W'	W'	W'	W'	W'
Widely used properties	TMHMM-7helices	W'	W'	W'	W'	W'	W'	W'	W'
Widely used properties	Phosphorylation	W'	W'	W'	W'	W'	W'	W'	W'
Widely used properties	Phosphorylation-S	W'	W'	W'	W'	W'	W'	W'	W'
Widely used properties	Phosphorylation-Y	W'	W'	W'	W'	W'	W'	W'	W'
Widely used properties	Phosphorylation-T	W'	W'	W'	W'	W'	W'	W'	W'
Widely used properties	Ubiquitination	W'	W'	x	W'	x	W'	x	W'(partially)
Widely used properties	Acetylation	W'	W'	W'	W'	W'	W'	W'	W'
Newly proposed properties	Essential	N'	N'	N'	N'	N'	N'	N'	N'
Newly proposed properties	Expression level	N'	N'	N'	N'	N'	N'	N'	x
Newly proposed properties	Tissue specificity	N'	N'	N'	N'	N'	N'	N'	N'
Newly proposed properties	PD>=2	N'	N'	N'	N'	N'	N'	N'	N'
Newly proposed properties	SABLE	N'	N'	N'	N'	N'	N'	N'	N'
Newly proposed properties	SABLE&phospolylation	x	x	x	x	x	x	x	x
Newly proposed properties	SABLE&phospolylation(s)	x	x	x	x	x	x	x	x
Newly proposed properties	SABLE&phospolylation(T)	x	x	x	x	x	x	x	x
Newly proposed properties	SABLE&phospolylation(Y)	x	x	x	x	x	x	x	x
Newly proposed properties	SABLE&Acetylation	x	x	x	x	x	x	x	x
Newly proposed properties	SABLE&ubiqitination	x	x	x	x	x	x	x	x
