# Supplementary material for: In silico re-identification of properties of drug target proteins
Source: BMC Bioinformatics. 2017 May 31;18(Suppl 7):248. doi: 10.1186/s12859-017-1639-3 (PMC5471946; doi:10.1186/s12859-017-1639-3)
Supplement: Supplementary file 9 — Figure S7. 10-fold and 10x10-fold cross-validations result in terms of the F-score and the standard derivation. (A) 10-fold cross-validation for SVM. (B) 10-fold cross-validation for RF. (C) 10X10 fold cross-validation for SVM. (D) 10X10 fold cross-validation for RF. (PDF 207 kb) [file 12859_2017_1639_MOESM9_ESM.pdf]

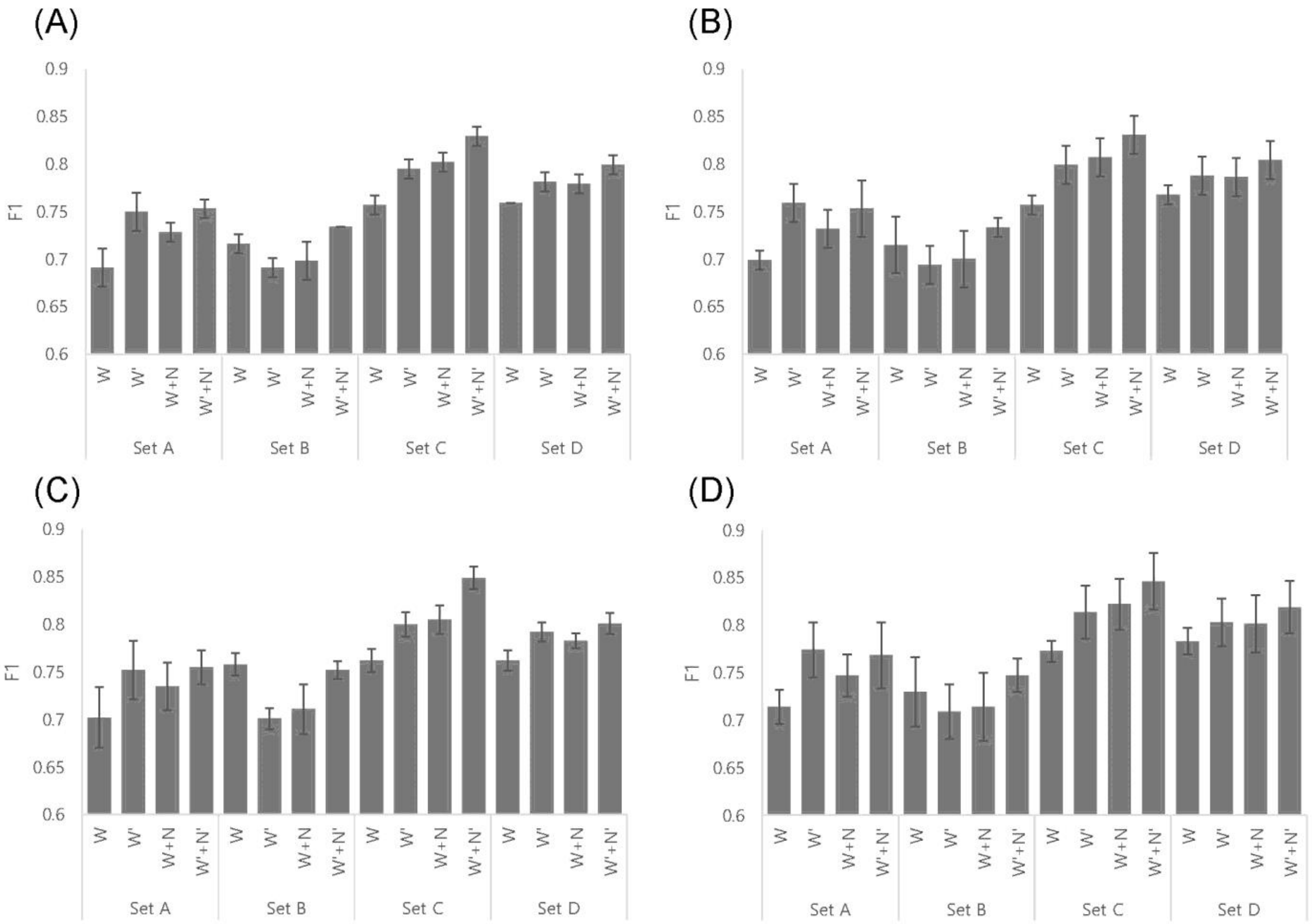

Supplementary Figure 7. 10-fold cross-validation and 10X10 cross-validation with standard derivation. (A) 10-fold cross-validation for SVM. (B) 10-fold cross-validation for RF. (C) 10X10 fold cross-validation for SVM. (D) 10X10 fold cross-validation for RF.
